# Serum-Free Medium Supplemented with *Haematococcus pluvialis* Extracts for the Growth of Human MRC-5 Fibroblasts

**DOI:** 10.3390/foods13183012

**Published:** 2024-09-23

**Authors:** Eun-Jeong Koh, Seong-Yeong Heo, Areumi Park, Yeon-Ji Lee, Woon-Yong Choi, Soo-Jin Heo

**Affiliations:** 1 Jeju Bio Research Center, Korea Institute of Ocean Science and Technology (KIOST), Jeju 63349, Republic of Korea; kej763@kiost.ac.kr (E.-J.K.); syheo@kiost.ac.kr (S.-Y.H.); areumi1001@kiost.ac.kr (A.P.); leeyj0409@kiost.ac.kr (Y.-J.L.); cwy@kiost.ac.kr (W.-Y.C.); 2 Department of Marine Technology & Convergence Engineering (Marine Biotechnology), University of Science and Technology, Daejeon 34113, Republic of Korea

**Keywords:** fetal bovine serum, *Haematococcus pluvialis*, cell proliferation, cell cycle progression, cyclin A/cyclin-dependent kinases 1 (CKD1)

## Abstract

Experiments are increasingly performed in vitro; therefore, cell culture technology is essential for scientific progress. Fetal bovine serum (FBS) is a key cell culture supplement providing growth factors, amino acids, and hormones. However, FBS is not readily available on the market, has contamination risks, and has ethical concerns. This study aimed to investigate *Haematococcus pluvialis* extracts (HE) as a potential substitute for FBS. Therefore, we assessed the effects of HE on cell maintenance, growth, and cycle progression in human lung fibroblasts (MRC-5). Cell progression and monosaccharide, fatty acid, and free amino acid compositions were analyzed using cell cycle analysis, bio-liquid chromatography, gas chromatography, and high-performance liquid chromatography, respectively. The results of nutritional profiles showed that the extracts contained essential amino acids required for synthesizing non-essential amino acids and other metabolic intermediates. Furthermore, most of the components present in HE were consistent with those found in FBS. HE enhanced cell viability and regulated cell cycle phases. Additionally, the interaction between growth factor cocktails and HE significantly improved cell viability, promoted cell cycle progression, and activated key cell cycle regulators, such as cyclin A and cyclin-dependent kinases 1 (CDK1). Our findings suggest that HE have considerable potential to substitute FBS in MRC-5 cell cultures and have functional and ethical advantages.

## 1. Introduction

Biological experiments involving living animals are increasingly performed in vitro; therefore, cell culture technology is important to science and technology. Notably, cell culture techniques are essential for in vitro experiments in cellular and molecular biology and biotechnology [1].

Most in vitro experiments using animal cells require culture medium and supplements for cell growth. Notably, fetal bovine serum (FBS) is a culture media supplement comprising numerous ingredients, including growth factors, essential amino acids, and hormones. FBS is primarily used in animal cell cultures because it is essential for cell attachment and maintenance [2]. FBS contains components that affect cell adhesion, such as fibronectin, laminin, and albumin, which activates the spreading and adhesion factors. Additionally, FBS contains cytokines and growth factors, such as insulin-like (IGF), platelet-derived (PDGF), and epidermal growth factors (EGF), affecting cell cycle progression and, subsequently, cell growth and differentiation [3].

Cell cycle progression is crucial for cell division and the production of daughter cells, which facilitate cell proliferation [4], and comprises four phases: G1 (cell growth), S (DNA synthesis), G2 (preparation for mitosis), and M (mitosis) [5]. Additionally, several genes, including cyclins and cyclin-dependent kinases (CDKs), regulate the four cell cycle phases. Cyclin D/CDK4/6 and cyclin A/CDK2 complexes are activated during the G1 and S phases, respectively. In the G2 phase, cyclin A/cyclin-dependent kinases 1 (CDK1) facilitates the transition from G2 to M, consistent with a previous study that revealed that the formation of Cdk1/cyclin B complexes initiated mitosis [4].

Therefore, FBS is a universal supplement; thus, its demand is increasing, leading to shortages. Furthermore, FBS is easily contaminated by prion proteins and has ethical issues associated with the killing of bovine fetuses to obtain FBS [6]. Therefore, there is a need to find alternatives to FBS.

Previous studies used serum-free media supplemented with marine materials, which contain abundant nutrients to maintain cell growth [7,8]. In particular, microalgae are utilized in the nutraceutical, pharmaceutical, and agricultural industries because of their various advantages, such as environmental friendliness, being produced sustainably, and high nutrient content [9]. A study has reported that microalgae contain amino acids and monosaccharides, with glutamate, which is an essential component in synthetic media, being particularly abundant in the algae [7]. In addition, previous studies have shown that microalgal extracts enhance cell viability and promote cell cycle progression by stimulating the G2/M phase in mammalian cells such as H460 and embryonic bovine tracheal (EBTr) fibroblast, compared to serum-free media [10,11]. Therefore, microalgae can substitute FBS as supplements for stem cell proliferation and differentiation [10,12].

*Haematococcus pluvialis* is a freshwater microalga known for its high content of astaxanthin, a potent antioxidant naturally found in microalgae, fish, and crustaceans. Due to its nutritional and functional properties, *H. pluvialis* has significant potential for various industrial applications, including dietary supplements, pharmaceuticals, and biofuels [13]. Nutrient profile analyses have revealed that this algae contains protein (21–23%), carbohydrates (63%), and lipids (40%) [14]. Notably, 46% of its total amino acid content consists of essential amino acids, with glutamic and aspartic acids being the most abundant. Additionally, glucose constitutes the highest proportion of carbohydrates, and palmitic, linoleic, and linolenic acids are in significant amounts in the total fatty acid profile of *H. pluvialis.* Although several studies have demonstrated the beneficial effects of *Haematococcus* on anti-inflammation, anti-oxidant properties, and anti-cancer activities, research demonstrating its potential as an alternative to FBS remains limited [15].

In this study, we investigated the effects of *H. pluvialis* extracts (HE) on cell maintenance and growth and elucidated the mechanisms of cell cycle progression. Furthermore, we evaluated *H. pluvialis* extracts (HE) potential to substitute FBS in in vitro experiments.

## 2. Materials and Methods

### 2.1. Materials

The materials and reagents used in the study included *H. pluvialis* powder (Gaussdream, Gyeonggi, Republic of Korea), Trypsin-ethylenediaminetetraacetic acid (0.25% (Sigma-Aldrich, St. Louis, MI, USA)), OptiPRO serum-free-media (SFM (Thermo Fisher Scientific, Waltham, MA, USA)), Dulbecco Modified Eagle Medium (DMEM (Thermo Fisher Scientific)), Quanti-Max WST-8 Cell Viability Assay Kit (BIOMAX, Gyeonggi, Republic of Korea), Growth factor (GFs (EGF, FGF-2, and IGF-1/PeproTech, Cranbury, NJ, USA)). Other materials include insulin, transferrin, and selenium growth factor (ITS), PDGF-BB, and transforming growth factor beta 1 (Sigma Aldrich, St Louis, MO, USA). Unless noted, all chemicals were purchased from Sigma-Aldrich, St. Louis, MO, USA.

### 2.2. Preparation of H. pluvialis Extracts (HE)

The *H. pluvialis* extracts (HE) were extracted using water at 90 °C for 6 h. Next, the H was concentrated in a vacuum evaporator (EYELA, Gyeonggi, Republic of Korea, and the concentrates were lyophilized for 48 h using a freezer (OPERON, Gyeonggi, Republic of Korea).

### 2.3. Determination of the Composition of Monosaccharides in HE

The monosaccharides in HE were analyzed at the Sejong University, Carbohydrate Bioproduct Research Center, Seoul, Republic of Korea. The extracts were hydrolyzed with 2 M trifluoroacetic acid at 100 °C for 2 h. The monosaccharide (galactose, glucose, fucose, arabinose, and rhamnose) contents were determined using Bio-liquid chromatography (LC) (Bio-LC system, Dionex, Sunnyvale, CA, USA) coupled with high-performance anion-exchange chromatography pulsed amperometric detection using a CarboPac PA1 anion-exchange column (4.0 × 50 mm (Thermo Fisher Scientific)). For the mobile phase, 18 mM NaOH/200 mM NaOH at 1 mL/min flow rate and 20 μL of samples were injected. 

### 2.4. Determination of Fatty Acid Components of HE

The fatty acid composition was analyzed at the National Instrumentation Center for Environmental Management (NICEM), Seoul, Republic of Korea, using the method described by Garces and Mancha [16]. Samples were extracted with methylation mixture (MeOH: Benzene:2,2-Dimethoxy-propane: H_2_SO_4_ = 3 9: 20: 5: 2]) at 80 °C for 2 h. Supernatants of the extracts were separated at room temperature. Fatty acids in the supernatant were analyzed using a gas chromatograph (Agilent 7890A, Agilent Technologies, Santa Clara, CA, USA) equipped with a flame ionization detector (280 °C, H_2_ 35, air 350, and He 35 mL/min (Agilent Technologies)) and a DB-23 column (Agilent Technologies, 60 mm × 0.25 mm × 0.25 μm). 

### 2.5. Determination of the Composition of Free-Amino Acid in HE

Free amino acid composition was determined at the NICEM. The sample was extracted using a buffer containing 0.1 M perchloric acid + 0.1% metaphosphoric acid in deionized water with sonication and shaking for 2 h. Extracts were filtered with 0.20 μm of filter and then analyzed using high-performance LC (Dionex Ultimate 3000, Thermo Fisher Scientific) with a fluorescence 1260 FLD (Agilent Technologies) and ultraviolet detectors using Inno C18 columns (4.6 mm × 150 mm, 5 um (YoungJin biochrom, Gyeonggi, Republic of Korea)). 

### 2.6. Cell Culture 

Human lung fibroblasts (MRC-5) were purchased from the Korean Cell Line Bank, Seoul, Republic of Korea. MRC-5 cells were grown in DMEM media containing 1% antibiotic antimycotic solution and 10% FBS at 37 °C in 5% CO_2_. For subsequent experiments, the MRC-5cells were replaced with serum-free medium (DMEM (N), control group) or serum-free medium supplemented with 5 ng/mL of the cocktail of growth factors (GFs) comprising 50, 100, and 200 μg/mL HE or SFM for 24 and 48 h. The cocktail (GFs) and *H. pluvialis* extracts (HE) were dissolved in phosphate-buffered saline (PBS).

### 2.7. Cell Viability and Proliferation 

MRC-5 cells (2 × 10^3^ cells/well) were seeded in 96 well plates. Cells were replaced with serum-free media (DMEM (N), control group) or serum-free medium supplemented with 5 ng/mL of a cocktail of growth factors (GFs) or 50, 100, and 200 μg/mL HE or SFM for 24 and 48 h. Next, 10% CCK-8 solution (Dojindo Laboratories, Kumamoto, Japan) was added to the well plates and incubated for 2 h. The absorbance was determined at 450 nm using a microplate reader (BioTek, Winooski, VT, USA).

### 2.8. Cell Cycle Analysis 

Cell cycle analysis was performed using a flow cytometer (BD Accuri C6, BD, Franklin Lakes, NJ, USA) to assess DNA fragmentation in cells using propidium iodide (PI) staining (Sigma-Aldrich). MRC-5 cells (1 × 10^5^ cells/well) were seeded into a 60 mm dish. Cells were replaced with serum-free media or serum-free medium supplemented with 5 ng/mL of a cocktail of growth factors: 50, 100, and 200 μg/mL H or SFM for 24 h. Cells were harvested, washed in PBS, fixed in 70% ethanol at −20 °C for 1 h, rinsed thrice in PBS, and stained with PI/RNase for 1 h at 36 °C. 

### 2.9. Quantitative Real-Time Polymerase Chain Reaction

Total RNAs were extracted from MRC-5 cells using the TRIzol reagent (Thermo Fisher Scientific) and reverse transcribed into cDNA using a thermocycler (iNtRON Biotechnology, Gyeonggi, Republic of Korea). Quantitative real-time polymerase chain reaction was performed using a PowerUp SYBR Green Master Mix (Thermo Fisher Scientific) on a thermal cycler dice real-time system III (TaKaRa, Tokyo, Japan). The PCR amplification conditions consisted of 30 s at 95 °C followed by 40 cycles of the denaturation step at 95 °C for 30 s and annealing and extension for 1 min at 60 °C. The relative mRNA expression of target genes was calculated using the 2^−ΔΔCt^ method and normalized to GAPDH as an internal reference gene. All primer sequences were designed using the National Center for Biotechnology Information BLAST and are listed in Table 1.

### 2.10. Statistical Analysis

All statistical analyses were performed using the Statistical Package for Social Sciences (version 12.0; (SPSS Inc., Armonk, NY, USA)). One-way analysis of variance was used to compare groups. Significant differences between mean values were assessed using Duncan’s test. The *p*-value in the multiple comparison results (* and **) indicate significant differences among the groups (*p* < 0.05 and *p* < 0.01, respectively).

## 3. Results

### 3.1. Nutritional Composition of HE

We analyzed the monosaccharides, fatty acids, and amino acids in HE. The results revealed that HE contained 19.52% galactose, 14.08% glucose, and three other monosaccharides (Table 2). Additionally, the extracts had 14 fatty acids, with palmitic acid being the most abundant at 4% (Table 3). HE contained 13,579 mg/kg glutamic, 10,669 mg/kg aspartic, and 16 other free amino acids (Table 4). Furthermore, the microalgal extracts contained more free amino acids than monosaccharides and fatty acids, suggesting that HE improved the growth of MRC-5 cells.

### 3.2. Effects of Serum-Free Media Supplemented with HE on Cell Proliferation 

**Figure 1:**
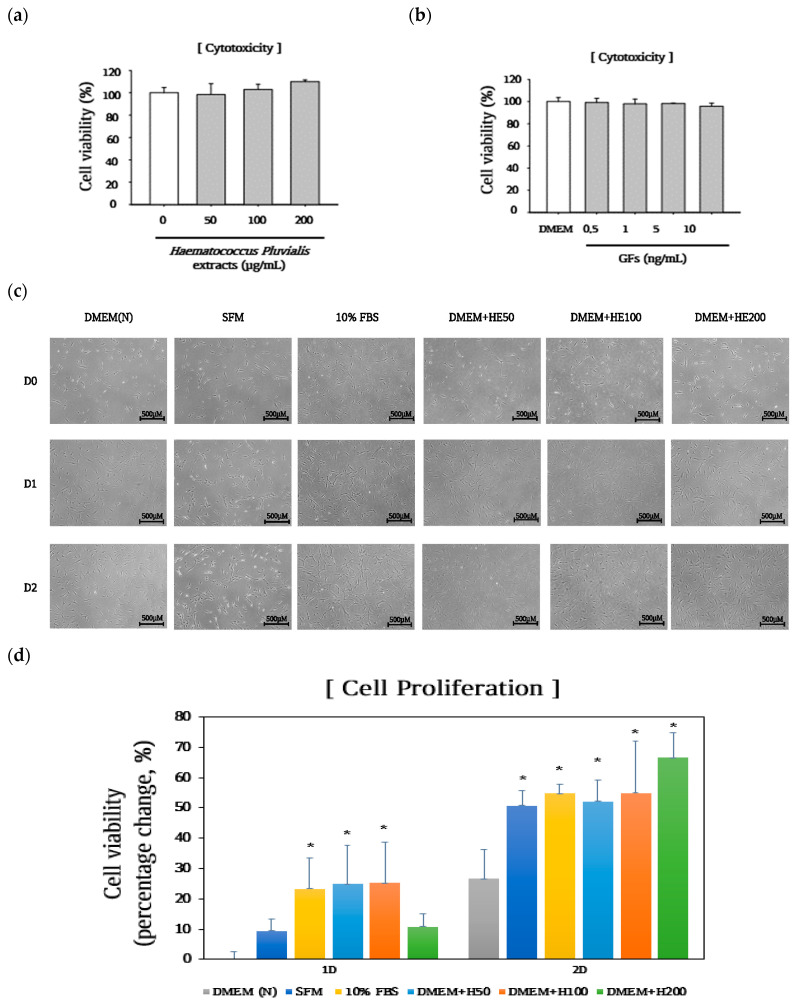
Effects of serum-free media supplemented with *Haematococcus pluvialis* extracts (HE) on cell proliferation after 48 h. (**a**,**b**) MRC-5 cells treated with HE and growth factor cocktails for 24 h. (**c**) Cell morphology and (**d**) proliferation after 48 h of treatment. The *p*-value in the multiple comparison results (*) indicates significant differences among the Dulbecco Modified Eagle Medium groups (* *p* < 0.05). Dulbecco Modified Eagle Medium (DMEM(N)), serum-free media; SFM, OptiPROserum-free-media; FBS, 10% Fetal bovine serum; DMEM + H50, Dulbecco Modified Eagle Medium + 50 μg/mL HE; DMEM + HE100, Dulbecco Modified Eagle Medium + 100 μg/mL HE; DMEM + HE200, Dulbecco Modified Eagle Medium + 200 μg/mL HE.

MRC-5 cells were treated with up to 200 μg/mL extract and 10 ng/mL of GFs to determine the optimal concentration of HE and GFs. Cell viability was analyzed using the WST-8 assay after 24 h. The extracts and GFs were not toxic to the treated cells (Figure 1a,b). Therefore, this study used concentrations of up to 200 μg/mL for the extracts (HE) and 10 ng/mL for the GFs. Next, to assess the proliferative effects of serum-free media supplemented with HE, cells were incubated in serum-free media with up to 200 μg/mL extracts, and cell morphology and cell viability were measured after 24 and 48 h. As described in Figure 1c, HE elevated cell density without altering cell morphology compared with the DMEM group. Additionally, the results revealed that the cell viability gradually increased in DMEM without FBS (DMEM (N), 26.6%) compared with that in the FBS group (FBS, 54.6%) after 48 h. However, SFM (SFM, 50.7%) and HE (HE200, 66.4%) significantly elevated cell viability compared with DMEM (*p* < 0.05) at 48 h.

**Figure 2:**
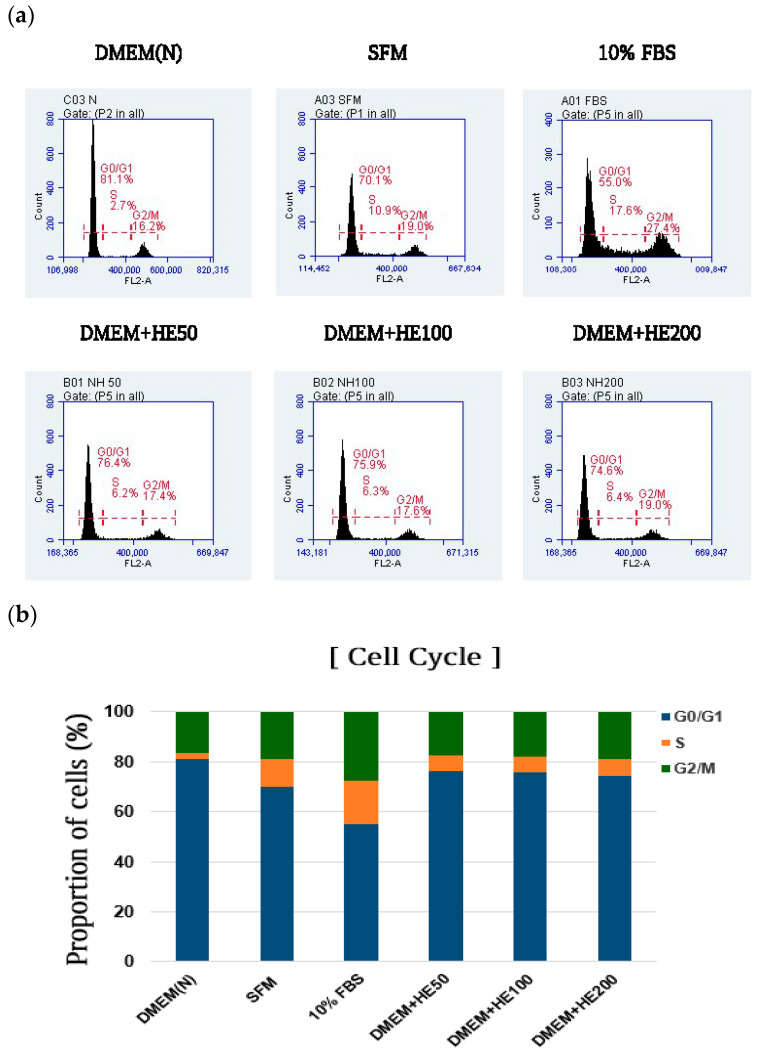
Effects of serum-free media supplemented with *Haematococcus pluvialis* extracts (HE) on cell cycle progression. (**a**) Distribution of cell cycle presented as flow cytometry histograms of MRC-5 cells (**b**) Cell cycle distribution of the total cell proportion. Dulbecco Modified Eagle Medium (DMEM(N)), serum-free media; SFM, OptiPROserum-free-media; FBS, 10% Fetal bovine serum; DMEM + H50, Dulbecco Modified Eagle Medium + 50 μg/mL HE; DMEM + HE100, Dulbecco Modified Eagle Medium + 100 μg/mL HE; DMEM + HE200, Dulbecco Modified Eagle Medium + 200 μg/mL HE.

Moreover, cells treated with serum-free media supplemented with HE were analyzed using flow cytometry to investigate their effects on cell cycle progression. Cells cultured in DMEM without serum increased in the G0/G1 phase (81.1%) and decreased during the G2/M phase (16.2%) (Figure 2a,b). Contrastingly, the media containing 10% FBS decreased the proportion of cells in the G0/G1 phase (55.0%) and increased that in the G2/M phase (27.4%) compared with that of the DMEM group. Additionally, MRC-5 cells incubated in serum-free media with HE decreased and increased in the G0/G1 phase (74.6%) and G2/M phase (19.0%), respectively, in a dose-dependent manner compared with those in the DMEM group, indicating that supplementation with HE in serum-free media activated cell cycle progression, which was delayed in cells cultured in DMEM without 10% FBS.

### 3.3. Effects of Serum-Free Media Supplemented with GFs on Cell Proliferation

**Figure 3:**
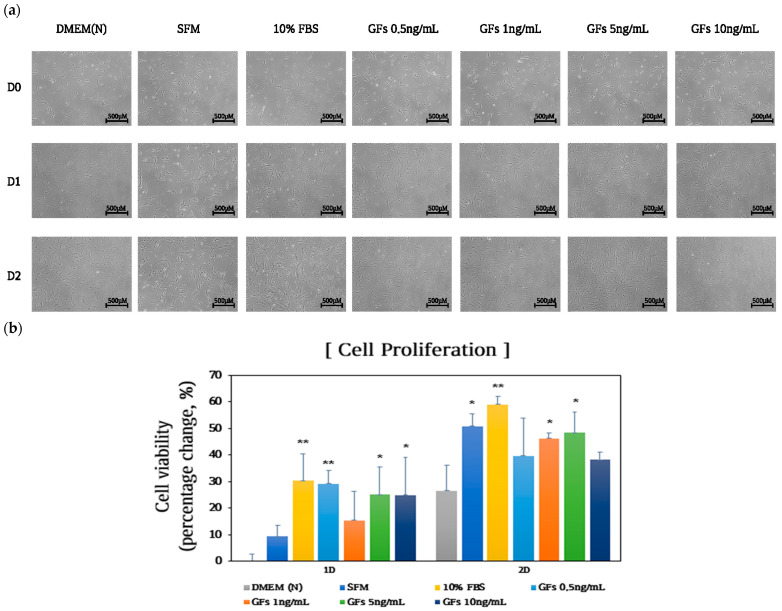
Effects of Serum-free media supplemented with growth factor cocktails on cell proliferation. (**a**) Cell morphology (**b**) and proliferation 48 h after treatment. The *p*-value in the multiple comparison results (*, **) indicates significant differences among the Dulbecco Modified Eagle Medium groups (* *p* < 0.05, ** *p* < 0.01). Dulbecco Modified Eagle Medium (DMEM(N)), serum-free media; SFM, OptiPRO serum-free-media; FBS, 10% fetal bovine serum; GFs, growth factor cocktails.

The results exhibited that GFs gradually increased cell proliferation without causing morphological changes at 24 h after treatment compared with the DMEM group (Figure 3a). However, cells treated with 10 ng/mL GFs did not exhibit proliferation after 48 h. Moreover, cell viability increased by 48.2% with the addition of up to 5 ng/mL GFs compared with that of the DMEM group (*p* < 0.05) at 48 h. Contrastingly, treatment with 10 ng/mL GFs reduced cell viability by 38.1% (Figure 3b) compared with 5 ng/mL GFs. 

**Figure 4:**
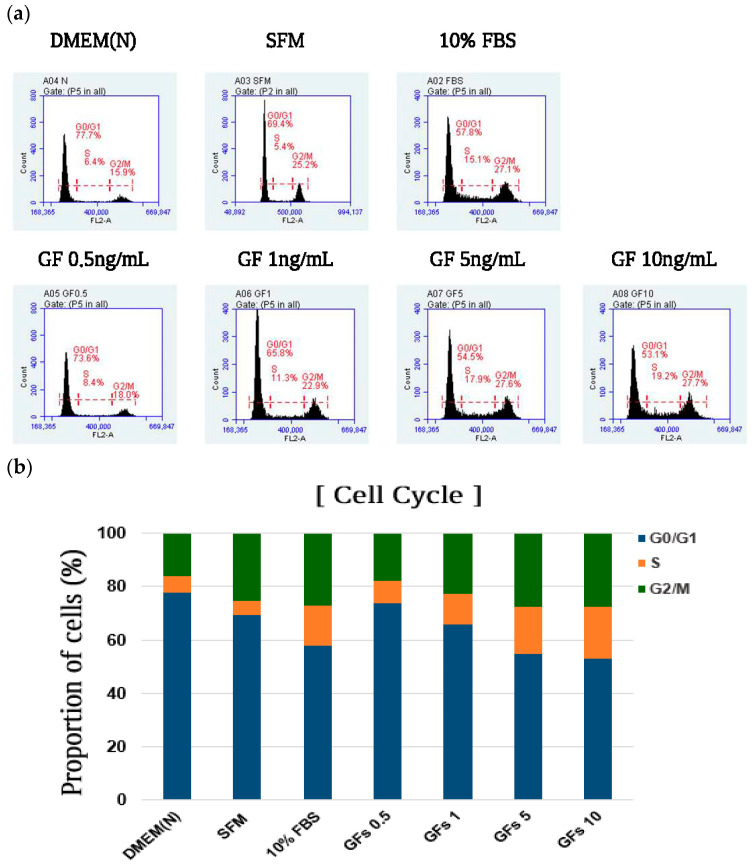
Effects of serum-free media supplemented with growth factor cocktails on the distribution of cell cycle. (**a**) Distribution of cell cycle presented as flow cytometry histograms of MRC-5 cells (**b**) Quantification of the cell cycle distribution of the total cell population. Dulbecco Modified Eagle Medium (DMEM(N)), serum-free media; SFM, OptiPRO se-rum-free-media; FBS, 10% fetal bovine serum; GFs, growth factor cocktails.

The cells were analyzed using PI staining and fluorescence-activated cell sorting (FACS) to examine the effects of GFs on the cell cycle. The proportion of cells in the G0/G1 phase increased to 77.7% in the DMEM (N) group compared with 57.8% in the 10% FBS group (Figure 4a,b). Conversely, the proportion of cells in the G2/M phase decreased in the serum-free medium group (15.9%) compared with that in the 10% FBS group (27.1%). Additionally, treatment with approximately 10 ng/mL GFs decreased and increased the proportion of cells in the G0/G1 and G2/M phases to 53.1% and 27.7%, respectively, indicating that GFs promote cell proliferation by activating cell cycle progression. Considering the decrease in cell proliferation at 10 ng/mL, a concentration of 5 ng/mL GFs was used for growth factor treatment in subsequent experiments. 

### 3.4. Effects of Serum-Free Media Supplemented with GFs and HE on Cell Growth and Cell Cycle Progression

**Figure 5:**
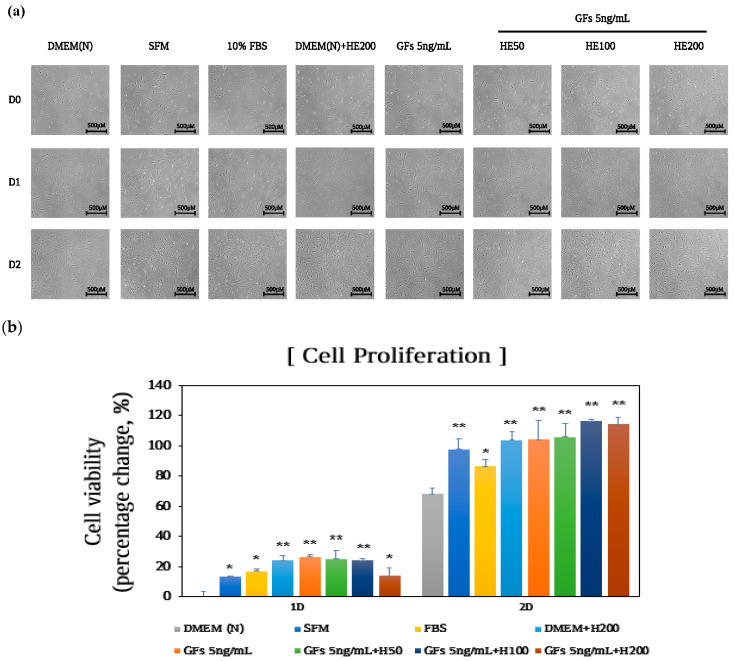
Effects of serum-free media supplemented with growth factor cocktails and *Haematococcus pluvialis* extracts (HE) on cell growth. (**a**) Cell morphology and (**b**) proliferation after 48 h of treatment. The *p*-value in the multiple comparison results (*, **) indicates significant differences among the Dulbecco Modified Eagle Medium groups (* *p* < 0.05, ** *p* < 0.01) Dulbecco Modified Eagle Medium (DMEM(N)), serum-free media; SFM, OptiPRO serum-free media; FBS, 10% fetal bovine serum; GFs, growth factor cocktails; GFs + HE50, growth factor cocktail and 50 μg/mL HE; GFs + HE100, growth factor cocktail and 100 μg/mL HE; GFs + HE200, growth factor cocktail and 200 μg/mL HE.

To determine the synergistic effects of GFs and HE, cells were treated with 5 ng/mL Gfs and up to 200 μg/mL HE, and cell morphology viability was determined over 48 h. A mixture of 5 ng/mL GFs and up to 200 μg/mL HE increased cell proliferation without altering cell morphology (Figure 5a,b). Furthermore, cell viability was significantly elevated in the mixture of GFs and up to 200 μg/mL HE compared with that in the DMEM group (*p* < 0.01). Notably, the combination of 200 μg/mL extracts and 5 ng/mL GFs (GFs 5 ng/mL + HE200) increased cell growth by 114.3% compared with 104.1% recorded in the treatment with 5 ng/mL GFs alone. 

**Figure 6:**
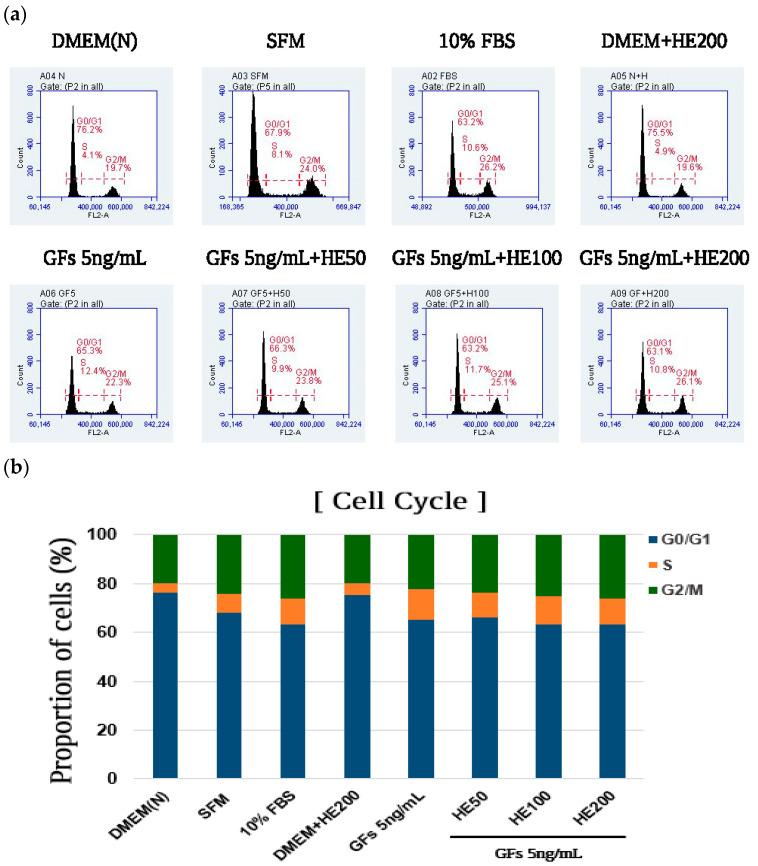
Effects of serum-free media supplemented with growth factor cocktails (GFs) and *Haematococcus pluvialis* extracts (HE) on cell cycle progression. (**a**) Distribution of cell cycle presented as flow cytometry histograms of MRC-5 cells (**b**) Quantification of the cell cycle distribution of the total cell population. Dulbecco Modified Eagle Medium (DMEM(N)), serum-free media; SFM, OptiPRO serum-free media; FBS, 10% fetal bovine serum; GFs, growth factor cocktails; GFs + HE50, growth factor cocktail and 50 μg/mL HE; GFs + HE100, growth factor cocktail and 100 μg/mL HE; GFs + HE200, growth factor cocktail and 200 μg/mL HE.

Moreover, the cells were analyzed to investigate cell cycle progression. GFs 5 ng/mL + HE200 reduced the proportion of cells in the G0/G1 phase by 63.1%, compared with those in the DMEM (N) (76.2%) group (Figure 6a,b). Additionally, GFs 5 ng/mL + HE200 increased the proportion of cells (26.1%) in the G2/M phase more than that observed in the DMEM (N) group (19.7%). The GFs and HE combination had a similar proportion of cells in the G0/G1 (63.2%) and G2/M (26.2%) phases compared with those of 10% FBS, demonstrating the synergistic effects of GFs and HE in stimulating cell cycle progression in the G0/G1 and G2/M phases. 

### 3.5. Effects of Serum-Free Media Supplemented with GFs and HE on Regulators of Cell Cycle Progression

**Figure 7:**
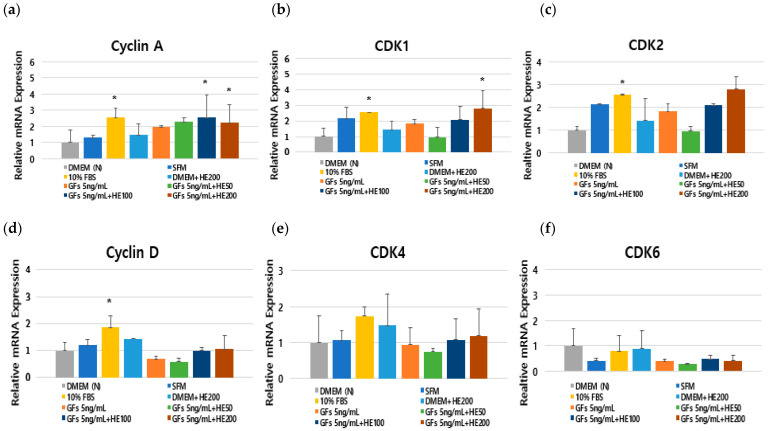
Effects of serum-free media supplemented with growth factor cocktails and *Haematococcus pluvialis* extracts (H) on cell cycle progression regulators. Expression of cyclin A (**a**), CDK1 (**b**), CDK2 (**c**), cyclin D (**d**), CDK4 (**e**), and CDK6 (**f**). The *p*-value in the multiple comparison results (*) indicates significant differences among the Dulbecco Modified Eagle Medium groups (* *p* < 0.05). Dulbecco Modified Eagle Medium (DMEM(N)), serum-free media; SFM, OptiPRO serum-free media; FBS, 10% fetal bovine serum; GFs, growth factor cocktails; GFs + HE50, growth factor cocktail and 50 μg/mL HE; GFs + HE100, growth factor cocktail and 100 μg/mL HE; GFs + HE200, growth factor cocktail and 200 μg/mL HE.

We analyzed the mRNA expression of cell cycle regulators (cyclin A and D, and Cdk1, 2, 4, and 6) to demonstrate how DMEM supplemented with GFs and HE activates cell cycle progression. Cell cycle regulators increased in cells incubated with medium containing 10% FBS compared with that containing serum-free medium (Figure 7). The GFs and HE combination significantly increased the mRNA levels of cyclin A and Cdk1 related to the G2/M phase, compared with that of the DMEM group (*p* < 0.05) (Figure 7a,c). Meanwhile, the expression of Cdk2 related to the S phase increased with treatment with a combination of GFs and HE. Additionally, the expression of Cyclin D, Cdk4, and Cdk6 genes, regulating the G0/G1 phase, increased with treatment with a combination of GFs and HE (Figure 7d,f), indicating that a mixture of GFs and HE activates cell cycle progression by enhancing cyclin A/Cdk1 expression, which is modulated during the G2/M phase.

## 4. Discussion

FBS is an essential supplement for culturing animal cells in biotechnological studies. However, its universal use is controversial due to issues related to cost, contamination, and ethics [1]. Therefore, various products from plants, bacteria, and marine organisms have been investigated as potential alternatives to FBS [2]. Marine biological resources have potential as substitutes as they are environmentally friendly and have high nutritional content [8,17]. Therefore, we screened numerous marine resources, including seaweeds and microalgae, to identify alternatives to FBS for culturing MRC-5 cells. The results indicated that HE was more beneficial for maintaining cell proliferation in MRC-5 cells grown without 10% FBS compared with other species. 

Based on the screening data, we identified potentially beneficial components of HE for cell maintenance and analyzed their composition. The results revealed that HE contained 5 monosaccharides, 14 fatty acids, and 18 amino acids. The most abundant components in the extracts were galactose (19%), glucose (14%), palmitic acid (4.1 mg/kg), and glutamic acid (13,580 mg/kg) (Table 2, Table 3 and Table 4).

FBS consists of numerous components such as serum, transport proteins, growth factors, fatty acids, and carbohydrates. A previous study demonstrated that commercial FBS contained 19 blood components, 30 free amino acids, and 11 fatty acids [18]. A previous study revealed that glucose (132 mg/dL), glutamic acid (61.5 g/L), and palmitic acid (745 mg/dL) were the most abundant in FBS [18].

Compared to the nutritional profiles, most of the components present in HE were consistent with those found in FBS. The extracts contained essential amino acids (histidine, threonine, arginine, tyrosine, valine, phenylalanine, isoleucine, leucine, and lysine) required for synthesizing non-essential amino acids and other metabolic intermediates [19].

The HE glucose content (14%) was lower than that of the DMEM medium (20%) [20]. However, HE contained higher essential amino (valine, leucine, isoleucine, threonine, phenylalanine, and lysine) and fatty acids than those in the DMEM group. As shown in Figure 1, cell growth in the DMEM group was delayed over 48 h compared with the FBS group. We considered that this reduction in cell proliferation may be attributed to the lack of components such as glutamic acid, aspartic acid, and alanine which are necessary for cell maintenance and proliferation. However, HE enhanced cell growth without altering cell morphology after 48 h more than that of serum-free medium owing to its abundant amino and fatty acid contents.

Additionally, cell proliferation is essential for cell maintenance, and modulation of cell cycle progression is crucial [4]. The DMEM without serum delayed cell cycle progression and increased the proportion of cells in the G0/G1 phase compared with that of the FBS. However, the FACS results indicated that the cell cycle was reactivated as HE addition reduced the proportion of cells in the G0/G1 phase and increased those in the G2/M phase.

Moreover, GFs ensure that cells develop through critical restriction points in the cell cycle [21]. GFs such as IGF-I, EGF, PDGF-AA, and PDGF-BB promote cell cycle entry >5-fold compared with that of serum-free media during the transition from the G0 to S phases [22]. We analyzed cell viability and cell cycle progression to elucidate the effects of GFs on cell proliferation in DMEM. The addition of GFs enhanced the cell cycle compared with the serum-free medium (Figure 3 and Figure 4). We used 5 ng/mL GFs because we observed a decrease in cell viability and changes in cell morphology at 10 ng/mL GFs after 48 h, demonstrating that the GFs cocktails stimulated cell cycle entry by passing the restriction point of the cell cycle, indicating that the GFs and HE combination had synergistic effects on cell proliferation by stimulating cell cycle entry (Figure 5, Figure 6 and Figure 7). Notably, HE regulated the cyclin A/Cdk1 complex related to the G2 phase. We hypothesized that the glutamic acid in HE promotes cell cycle expansion in cells grown in serum-free media.

Glutamate, which is abundant in red microalgae extracts and synthesized from glutamine, is a non-essential amino acid, a key bioenergetic substrate for proliferating cells, and an excitatory neurotransmitter [23]. Glutamate addition promotes cell cycle progression from G1 to S phases through to the G2/M phase [24]. Additionally, glutamate activates metabotropic glutamate receptors, which are protein-coupled receptors that regulate the cell cycle and promote cell proliferation through the AKT/mTORC1 signaling pathway [25]. Therefore, we suggest that GFs may quicken cell cycle entry from the G0 to S phases, and HE may enhance progression through the G2/M phase in MRC-5 cells. Given the abundance of glutamate in HE, we suggest that the glutamic acid in HE may stimulate cell cycle entry in the G2/M phase. However, this hypothesis could not be fully understood in this study. Future research should determine whether the glutamic acid in HE affects cell growth and elucidate the mechanisms related to the AKT/mTORC1 signaling pathway.

## 5. Conclusions

In conclusion, the addition of HE maintained cell morphology and growth in serum-free media and regulated cell cycle progression through the G2/M phases Moreover, the combination of GFs and HE had synergistic effects on cell cycle progression by activating cell cycle regulators, such as cyclin A and CDK1. Furthermore, considering that most of the components present in HE are consistent with those found in FBS, HE shows potential as an alternative to FBS in MRC-5 cell culture.

## Figures and Tables

**Table 1:** Sequence data of primers used in reverse-transcription quantitative polymerase chain reaction of specific genes.

Gene Name	Forward (5→3)	Reverse (5→3)
Cyclin A	GGTACTGAAGTCCGGGAACC	TGAACGCAGGCTGTTTACTG
Cyclin D	GGCGGAGGAGAACAAACAGA	CTCCTCAGGTTCAGGCCTTG
CDK1	CTGGGGTCAGCTCGTTACTC	GGAGTGCCCAAAGCTCTGAA
CDK2	TCAAGCTGCTGGATGTCATTCA	CAGTGAGAGCAGAGGCATCCAT
CDK4	AGTGTGAGAGTCCCCAATGG	CCTTGATCTCCCGGTCAGTT
CDK6	TGGAGACCTTCGAGCACC	CACTCCAGGCTCTGGAACTT
GAPDH	CAATGACCCCTTCATTGACC	GACAAGCTTCCCGTTCTCAG

**Table 2:** Monosaccharide composition of *Haematococcus pluvialis* extracts (HE).

Monosaccharide Composition (%)	*H. pluvialis* Extracts (HE)
Galactose	19.52
Glucose	14.08
Fucose	3.23
Arabinose	3.47
Rhamnose	0.40

**Table 3:** Fatty acid composition of *Haematococcus pluvialis* extracts (HE).

Fatty Acid Composition (mg/g)	*H. pluvialis* Extracts (HE)
Palmitic acid	4.136
Oleic acid	0.949
Behenic acid (docosanoic acid)	0.764
Linoleic acid	0.685
Alpha-linolenic acid	0.525
Myristic acid	0.327
Stearic acid	0.246
Arachidonic acid	0.170
Lignoceric acid	0.165
Palmitoleic acid	0.163
Lauric acid (dodecanoic acid)	0.119
Arachidic acid (icosanoic acid)	0.094
Gamma-linolenic acid	0.073
Eicosapentaenoic acid	0.070

**Table 4:** Amino acid composition of *Haematococcus pluvialis* extracts (HE).

Amino Acid Composition (mg/kg)	*H. pluvialis* Extracts (HE)
Glutamic acid	13,579
Aspatic acid	10,669
Leucine	7984
Isoleucine	5306
Alanine	7482
Valine	6540
Glycine	6296
Taurine	6128
Arginine	5827
Serine	5797
Threonine	5722
Phenylalanine	5695
Lysine	4742
Proline	4362
Ornithine	2853
Tyrosine	2569
Histidine	1242
Citrulline	372

## Data Availability

The original contributions presented in the study are included in the article, and further inquiries can be directed to the corresponding author.

## References

[1] Liu S., Yang W., Li Y., Sun C. (2023). Fetal bovine serum, an important factor affecting the reproducibility of cell experiments. Sci. Rep..

[2] Chelladurai K.S., Christyraj J.D.S., Rajagopalan K., Yesudhason B.V., Venkatachalam S., Mohan M., Vasantha N.C., Christyraj J.R.S.S. (2021). Alternative to FBS in animal cell culture-An overview and future perspective. Heliyon.

[3] Pilgrim C.R., McCahill K.A., Rops J.G., Dufour J.M., Russell K.A., Koch T.G. (2022). A review of fetal bovine serum in the culture of mesenchymal stromal cells and potential alternatives for veterinary medicine. Front. Vet. Sci..

[4] Wang Z. (2021). Regulation of cell cycle progression by growth factor-induced cell signaling. Cells.

[5] Heber-Katz E., Zhang Y., Bedelbaeva K., Song F., Chen X., Stocum D.L. (2013). Cell cycle regulation and regeneration. New Perspect. Regen..

[6] Lee S.Y., Yun S.H., Jeong J.W., Kim J.H., Kim H.W., Choi J.S., Kim G.-D., Joo S.T., Choi I., Hur S.J. (2022). Review of the current research on fetal bovine serum and the development of cultured meat. Food Sci. Anim. Resour..

[7] Dong N., Xue C., Yang Y., Chang Y., Wang Y., Guo H., Liu Y., Wang Y. (2023). Auxenochlorella pyrenoidosa extract supplementation replacing fetal bovine serum for Carassius auratus muscle cell culture under low-serum conditions. Food Res. Int..

[8] Amirvaresi A., Ovissipour R. (2024). Evaluation of Plant-and Microbial-Derived Protein Hydrolysates as Substitutes for Fetal Bovine Serum in Cultivated Seafood Cell Culture Media. BioRxiv.

[9] Chen C., Tang T., Shi Q., Zhou Z., Fan J. (2022). The potential and challenge of microalgae as promising future food sources. Trends Food Sci. Technol..

[10] Jeong Y., Choi W.-Y., Park A., Lee Y.-J., Lee Y., Park G.-H., Lee S.-J., Lee W.-K., Ryu Y.-K., Kang D.-H. (2021). Marine cyanobacterium Spirulina maxima as an alternate to the animal cell culture medium supplement. Sci. Rep..

[11] Defendi-Cho G., Gould T.M. (2023). In vitro culture of bovine fibroblasts using select serum-free media supplemented with Chlorella vulgaris extract. BMC Biotechnol..

[12] Ghosh J., Akiyama Y., Haraguchi Y., Yamanaka K., Asahi T., Nakao Y., Shimizu T. (2024). Proliferation of mammalian cells with Chlorococcum littorale algal compounds without serum support. Biotechnol. Prog..

[13] Mularczyk M., Michalak I., Marycz K. (2020). Astaxanthin and other nutrients from *Haematococcus pluvialis*—Multifunctional applications. Mar. Drugs.

[14] Oslan S.N.H., Tan J.S., Oslan S.N., Matanjun P., Mokhtar R.A.M., Shapawi R., Huda N. (2021). Haematococcus pluvialis as a potential source of astaxanthin with diverse applications in industrial sectors: Current research and future directions. Molecules.

[15] Jannel S., Caro Y., Bermudes M., Petit T. (2020). Novel insights into the biotechnological production of *Haematococcus pluvialis*-derived astaxanthin: Advances and key challenges to allow its industrial use as novel food ingredient. J. Mar. Sci. Eng..

[16] Ryu Y.-K., Lee W.-K., Choi W.-Y., Kim T., Lee Y.-J., Park A., Kim T., Oh C., Heo S.-J., Kim J.H. (2023). A novel drying film culture method applying a natural phenomenon: Increased carotenoid production by *Haematococcus* sp.. Bioresour. Technol..

[17] Willoughby R., Pomponi S.A. (2000). Quantitative assessment of marine sponge cells in vitro: Development of improved growth medium. In Vitro Cell. Dev. Biol. Anim..

[18] Yun S.H., Lee S.Y., Lee J., Joo S.T., Choi I., Choi J.S., Kim G.D., Lee J., Choi S.-H., Hur S.J. (2023). Analysis of commercial fetal bovine serum (FBS) and its substitutes in the development of cultured meat. Food Res. Int..

[19] Salazar A., Keusgen M., Von Hagen J. (2016). Amino acids in the cultivation of mammalian cells. Amino Acids.

[20] Dulbecco R., Freeman G. (1959). Plaque production by the polyoma virus. Virology.

[21] Jones S.M., Kazlauskas A. (2001). Growth factor-dependent signaling and cell cycle progression. Chem. Rev..

[22] Gross S.M., Rotwein P. (2016). Unraveling growth factor signaling and cell cycle progression in individual fibroblasts. J. Biol. Chem..

[23] Prickett T.D., Samuels Y. (2012). Molecular pathways: Dysregulated glutamatergic signaling pathways in cancer. Clin. Cancer Res..

[24] Matés J.M., Pérez-Gómez C., de Castro I.N., Asenjo M., Márquez J. (2002). Glutamine and its relationship with intracellular redox status, oxidative stress and cell proliferation/death. Int. J. Biochem. Cell Biol..

[25] Willard S.S., Koochekpour S. (2013). Glutamate, glutamate receptors, and downstream signaling pathways. Int. J. Biol. Sci..

